# Variational Mode Decomposition for Raman Spectral Denoising

**DOI:** 10.3390/molecules28176406

**Published:** 2023-09-02

**Authors:** Xihui Bian, Zitong Shi, Yingjie Shao, Yuanyuan Chu, Xiaoyao Tan

**Affiliations:** 1 State Key Laboratory of Separation Membranes and Membrane Processes, School of Chemical Engineering and Technology, Tiangong University, Tianjin 300387, China; 2 NMPA Key Laboratory for Technology Research and Evaluation of Drug Products, Shandong University, Jinan 250012, China

**Keywords:** Raman spectrum, denoising, variational mode decomposition, empirical mode decomposition, mode mixing

## Abstract

As a fast and non-destructive spectroscopic analysis technique, Raman spectroscopy has been widely applied in chemistry. However, noise is usually unavoidable in Raman spectra. Hence, denoising is an important step before Raman spectral analysis. A novel spectral denoising method based on variational mode decomposition (VMD) was introduced to solve the above problem. The spectrum is decomposed into a series of modes (uk) by VMD. Then, the high-frequency noise modes are removed and the remaining modes are reconstructed to obtain the denoised spectrum. The proposed method was verified by two artificial noised signals and two Raman spectra of inorganic materials, i.e., MnCo ISAs/CN and Fe-NCNT. For comparison, empirical mode decomposition (EMD), Savitzky–Golay (SG) smoothing, and discrete wavelet transformation (DWT) are also investigated. At the same time, signal-to-noise ratio (SNR) was introduced as evaluation indicators to verify the performance of the proposed method. The results show that compared with EMD, VMD can significantly improve mode mixing and the endpoint effect. Moreover, the Raman spectrum by VMD denoising is more excellent than that of EMD, SG smoothing and DWT in terms of visualization and SNR. For the small sharp peaks, some information is lost after denoising by EMD, SG smoothing, DWT and VMD while VMD loses fewest information. Therefore, VMD may be an alternative method for Raman spectral denoising.

## 1. Introduction

Raman spectroscopy is a vibrational spectrum that excites molecular motion by light and is used for chemical analysis of the sample by interpreting the interaction, which is used for non-destructive analytical tools [1,2,3]. It is extensively used for qualitative and quantitative characterization in the fields of food [4,5], diseases [6,7,8], biochemistry [9,10] and materials science [11,12,13]. For example, Dai et al. [4] identified the age of pericarpium citri reticulatae (PCR) products by Raman spectroscopy. Zhang et al. [7] recorded comprehensive molecular information about tumors in situ by Raman spectroscopy. However, due to the influence of thermal noise and dark current, noise inevitably exists in Raman spectra. Moreover, the Raman signal is inherently weak, making useful information susceptible to noise [14]. Therefore, it is important to remove noise from Raman spectra.

Several methods have been used for spectral denoising, such as Savitzky–Golay (SG) smoothing [15,16], Whittaker smoother (WS) [17], deep learning (DL) [18,19], discrete wavelet transformation (DWT) [20,21], and empirical mode decomposition (EMD) [22,23,24]. SG smoothing, also known as convolution smoothing, is a signal smoothing method based on polynomial approximation of local least squares proposed by Savitzky and Golay [15]. It is a weighted average method, emphasizing the role of the center point. The advantage of SG smoothing is that it does not have a delay, and it can handle data missed in short periods of time well. Luo et al. [3] applied SG smoothing as a comparison method to the Raman spectral denoising of cefuroxime axetil tablets and a mixture of pyrene and cyclodextrin, as well as the Raman image denoising of cefuroxime axetil and cervical cancer cells. The intensities of the peaks in the Raman spectrum denoised by SG smoothing partially decreased with the retained noise. Meanwhile, due to the difference in the change rate of the background and the Raman spectrum, there is an irregular deviation between the background and the Raman spectrum. For SG smoothing, it is only performed once for the signal denoising. Moreover, it is important to choose the window size for the denoising performance. The different widths of the moving window can produce different denoising effects for SG smoothing. A large window size may result in the loss of valid information while a small window size may remain noise in the spectrum [25]. WS is a signal smoothing method based on penalized least squares proposed by Whittaker. The program of WS is concise and it has the ability to adapt to boundaries automatically. Compared with SG smoothing, WS has a fast computation speed and is easy to implement [26]. However, WS is not widely used because it requires the intervals between adjacent collection points of the data be equal. Due to the highly flexible characteristic, DL has become a hot topic and also can be used for signal denoising [18,19,27]. Horgan et al. [18] proposed a comprehensive framework for high-throughput molecular imaging through Raman spectroscopy that supports deep learning. It can effectively reconstruct Raman characteristics from low signal-to-noise ratio (SNR) Raman spectra. In the denoising process of DL, a model is built between the noised signals and the pure signals of the same samples in the training set. Then, the new noised signal is taken as the input to the model and the denoised signal is obtained. Although excellent denoising ability was demonstrated, DL denoising requires a lot of training samples. Furthermore, sometimes it is difficult to obtain the pure signals.

DWT and EMD are both decomposition-based denoising methods, which decompose the original signal into a certain number of components with different frequencies. Compared with DL, the two methods do not need training samples and pure signals. For DWT denoising, the original signal was decomposed into a series of details and approximations. The high-frequency details are deleted and the denoised signal is reconstructed by inverse wavelet transform [28]. Dou et al. [29] applied DWT to the research of intelligent transportation system data denoising and compression. However, it is difficult to select the optimal parameters for DWT because of the large number of wavelet functions and decomposition scales. An adaptive method that does not require a defined basis as DWT, EMD was proposed by Huang et al. in 1998 [22,23,24]. EMD has been applied to Raman spectral denoising. Leon-Bejarano et al. [30] applied EMD as an adaptive parameterless signal processing method to Raman spectrum denoising of biological samples. However, EMD has mode mixing and an endpoint effect in the decomposition process.

To overcome the drawbacks of EMD, variational mode decomposition (VMD) was proposed by Dragomiretskiy et al. in 2014 [31]. VMD can determine the relevant frequency band adaptively and estimate the corresponding modes (uk) simultaneously in the decomposition process [32]. Compared with EMD, VMD has a solid mathematical theory and can improve mode mixing and the endpoint effect [33]. It has been extensively used for bearing fault diagnosis [34,35,36], pipeline leak detection [37,38], tidal analysis [39], and wind speed forecasting [40,41]. However, few studies use VMD for spectral denoising in analytical chemistry.

In this research, VMD is introduced for Raman spectral denoising. The original spectrum is decomposed into a series of uk by VMD. The noise modes are deleted and the remaining modes are reconstructed to obtain the denoised spectrum. The feasibility and effectiveness of the method are validated by two artificial noised signals and two actual Raman spectra of inorganic materials. EMD, SG smoothing and DWT are used to compare with the proposed method, and the denoising effect is verified by visualization and SNR.

## 2. Results and Discussion

### 2.1. Denoising of the First Artificial Signal

**Figure 1:**
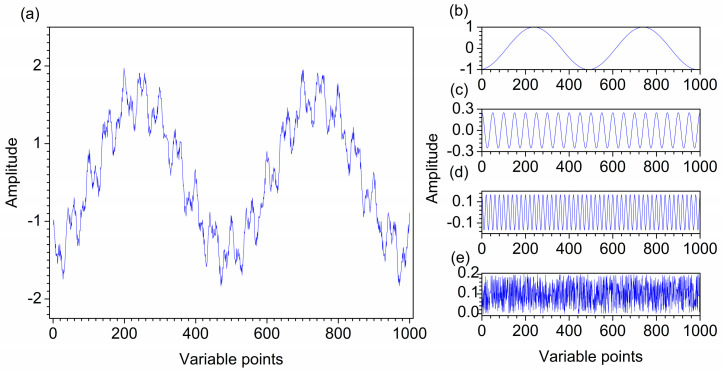
The first artificial noised signal (**a**) and its constituents f1 (**b**), f2 (**c**), f3 (**d**), and random noise (**e**).

**Figure 2:**
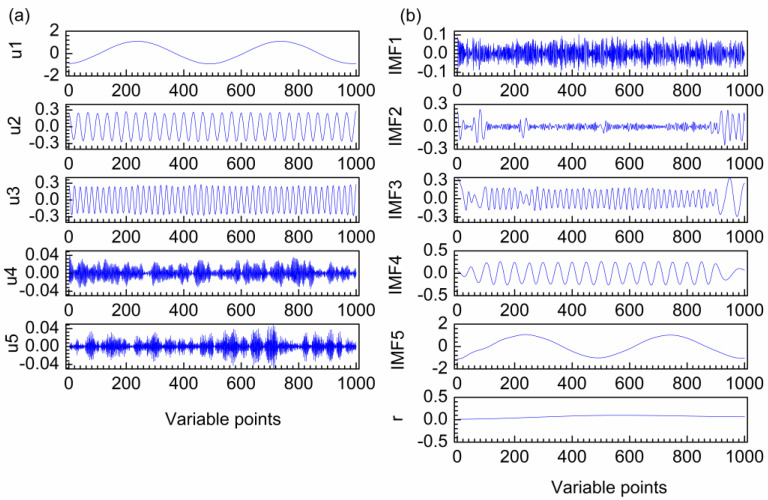
Decomposition results of VMD (**a**) and EMD (**b**) for the first artificial noised signal.

Figure 1a–e show the first artificial noised signal, the different cosine functions and random noise that construct the artificial signal. The frequency of the three functions from low to high, and the frequency of random noise is the highest. Ideally, the curves of b–d in Figure 1 can be clearly obtained after decomposition. Figure 2a shows the decomposition result of VMD for the first artificial noised signal, which is decomposed into five modes u1–u5. It can be seen that the frequency distribution of modes increases from low to high. The decomposition of VMD starts at the coarsest level. When the mode order increases, the oscillation frequencies of the modes also increase. The curve shapes of the three modes are almost the same as the three curves in Figure 1b–d, respectively. Obviously, the modes u1, u2 and u3 obtained by VMD correspond to the three functions f_1_, f_2_ and f_3_ that constitute the first artificial signal in Figure 1a. The modes u4 and u5 are random noise. Hence, the different frequency modes in the first artificial signal are separated completely by VMD.

The decomposition result of EMD is also investigated as shown in Figure 2b. Five intrinsic modal function components (IMFs) and a residual (**r**) are obtained. It is clear that the oscillation frequencies of the modes decrease as the order of IMFs increases. IMF2 and IMF3 are high frequency in the middle and low frequency at both ends, which are both high-frequency mixing modes. The pure signal of first artificial signal is consisted by three different frequency curves. However, it is decomposed to four low-frequency modes by EMD. Therefore, mode mixing and over-decomposition are produced for the first artificial signal by EMD.

**Figure 3:**
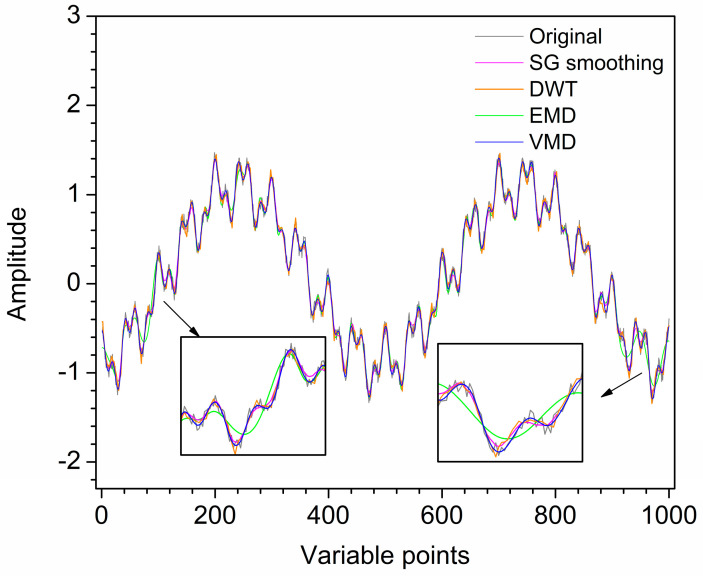
Denoised signals by different methods for the first artificial signal.

For further comparison, the first artificial noised signal is also denoised by SG smoothing and DWT. Figure 3 shows the denoised signals by SG smoothing, DWT, EMD and VMD. For SG smoothing, with window size 16, most noise has been removed. However, some peaks of the SG smoothing signal are deviated or lower than those of the original signal. For the result of DWT, the signal still contains noise, which makes each peak not smooth enough. Visual inspection of the decomposition result of EMD for the first artificial noised signal, IMF1 and IMF2 are the high-frequency modes which are removed as noise. IMF3–IMF5 and **r** are low-frequency modes, which are reconstructed to obtain the denoised signal. Due to IMF2 is a mixing mode, the signal denoised by EMD has an endpoint effect when IMF2 is removed, resulting in signal distortion at both ends. Furthermore, some information of the signal after denoising is lost due to mode mixing. For VMD, it is obvious that u1–u3 are low-frequency modes with useful information, and u4 and u5 are high-frequency modes with little useful information. Hence, the artificial denoised signal is obtained by reconstructing u1–u3, while u4 and u5 are removed as noise. Obviously, the denoising result of VMD improves mode mixing and endpoint effects generated by EMD. The denoised signal by VMD almost overlaps with the original signal. Hence, the denoising effect of VMD is ideal. The SNR values of SG smoothing, DWT, EMD and VMD are 22.11, 23.11, 19.95 and 23.31, respectively. Therefore, for the first artificial signal, VMD has superiority in visual inspection and SNR compared with SG smoothing, DWT and EMD.

### 2.2. Denoising of the Second Artificial Signal

**Figure 4:**
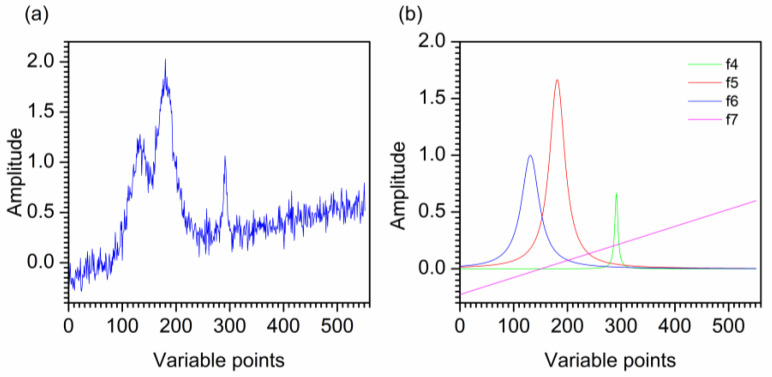
The second artificial noised signal (**a**) and its constituent curves (**b**).

**Figure 5:**
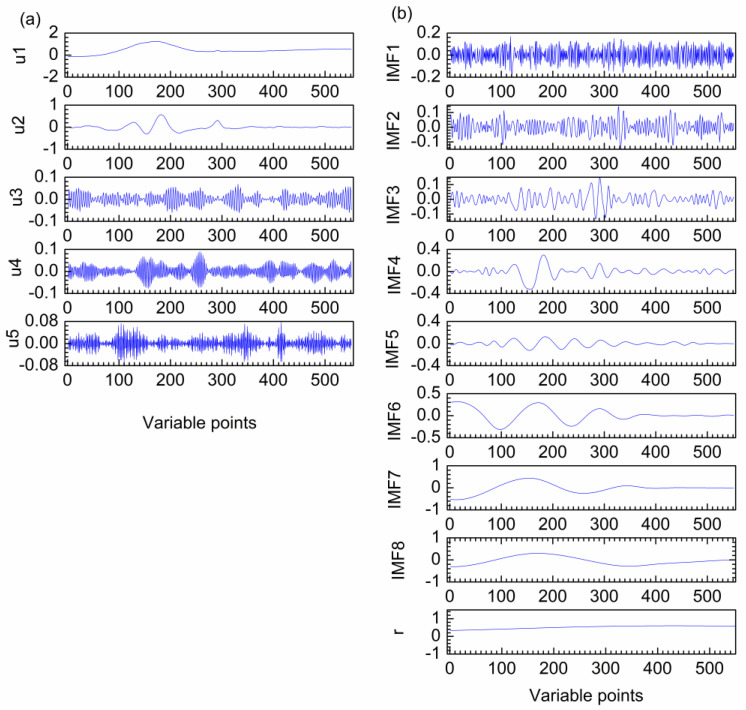
Decomposition results of VMD (**a**) and EMD (**b**) for the second artificial noised signal.

Figure 4a and b show the second artificial noised signal and its three constituent Lorentz functions and one line. The noised signal is decomposed by VMD, obtaining five modes u1–u5 as shown in Figure 5a. It can be seen that u1 and u2 are the low-frequency modes, u3–u5 are the high-frequency modes. The second artificial signal is effectively decomposed into different frequencies without mode mixing by VMD.

For comparison, the second artificial noised signal was also denoised by EMD. Figure 5b shows the decomposition result of EMD, obtaining IMF1–IMF8 and a **r**. IMF1–IMF3 are the high-frequency modes. IMF4–IMF8 and **r** are the low-frequency modes. However, the frequency of IMF4 is obviously low in the middle and relatively high at both ends, which indicates that IMF4 is a mixing mode. Hence, mode mixing is produced in the decomposition result of EMD.

**Figure 6:**
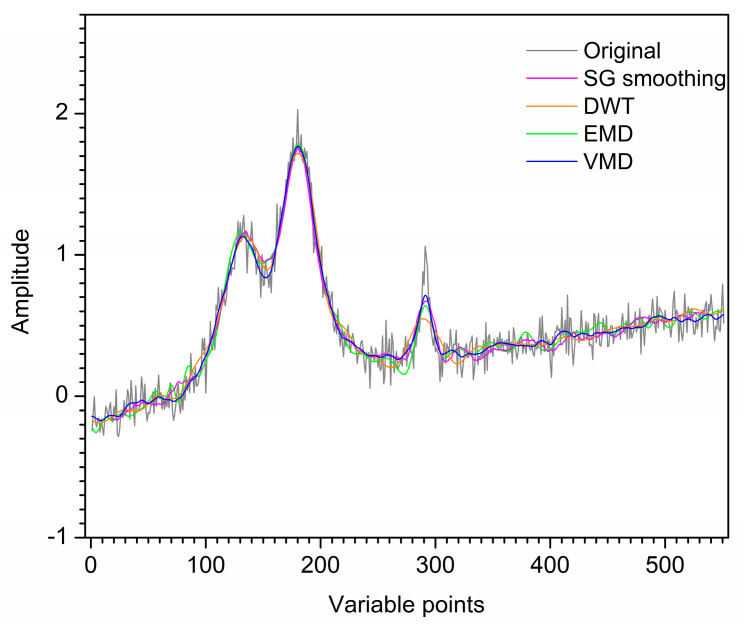
Denoised signals by different methods for the second artificial signal.

For the further comparison, SG smoothing and DWT are also applied to the second artificial noised signal. Figure 6 shows the denoised signals by SG smoothing, DWT, EMD and VMD. For SG smoothing, the window size is selected as 33. Visual inspection the decomposition result of EMD for the second artificial noised signal, IMF4–IMF8 and **r** are used to reconstruct the denoised signal. For the result of VMD, u1 and u2 are reconstructed to obtain the artificial denoised signal and u3–u5 are removed as noise. It is obvious that most noise has been removed by SG smoothing, DWT, EMD and VMD. The signal denoised by VMD is smoother than that of SG smoothing and EMD. For EMD, some useful information of the signal after denoising is lost due to mode mixing, resulting in signal distortion in the range of 110–190th points. As shown in Figure 6, the denoised signal has an endpoint effect at left end. Furthermore, the peaks at 120th and 190th points of the signal denoised by SG smoothing, EMD and VMD are basically coincident. However, the signal after SG smoothing still contains noise at the two peaks mentioned above. For the second artificial signal denoised by DWT, the peaks located at 120th and 190th points are lower than those of the original signal. Moreover, the valley around 150th point after SG smoothing, DWT and EMD deviate significantly from the original signal position. For the small sharp peak in the range of 290–310th points, some information is lost after denoising by the four denoising methods. However, SG smoothing, DWT and EMD lose more information than VMD denoising. Thus, VMD shows better denoising performance than SG smoothing, DWT and EMD.

In order to evaluate the denoising performance of the four methods more intuitively, the SNR as a criterion is calculated. The SNR values of SG smoothing, DWT, EMD and VMD are 15.43, 15.36, 16.3 and 16.8, respectively. Thus, VMD has superiority in SNR compared with SG smoothing, DWT and EMD.

### 2.3. Denoising of the Raman Spectrum of MnCo-ISAs/CN

**Figure 7:**
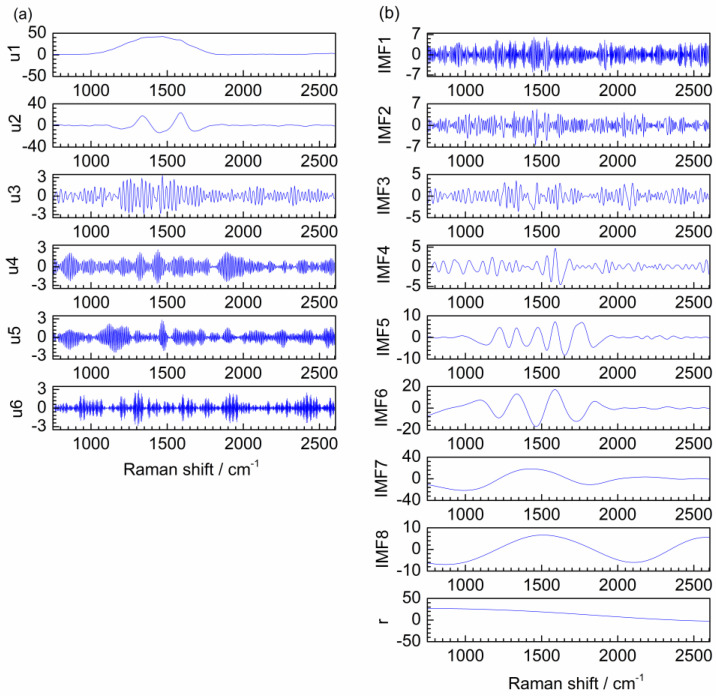
Decomposition results of VMD (**a**) and EMD (**b**) for the Raman spectrum of MnCo-ISAs/CN.

To evaluate the denoising effect of the proposed method on the actual spectrum, the Raman spectrum of MnCo single-atom catalyst anchored on carbon and nitrogen materials (MnCo-ISAs/CN) was used for denoising. Figure 7a shows the VMD decomposition result for Raman spectrum of MnCo-ISAs/CN, which composes of six modes u1–u6. Obviously, it is easy to distinguish the boundary of noise modes and useful information modes by visual inspection, u1 and u2 are the low-frequency modes and u3–u6 are the high-frequency modes.

For comparison, EMD is also applied to Raman spectrum denoising. The decomposition result of EMD is shown in Figure 7b, IMF1–IMF8 and a **r** are obtained. It is clear that IMF1–IMF3 are the high-frequency modes and IMF5–IMF8 and **r** are the low-frequency modes. However, IMF4 is low frequency in the range of 1400–1700 cm^−1^ and high frequency in other ranges. Thus, IMF4 is a mixing mode. Hence, mode mixing is produced in the decomposition result of EMD.

**Figure 8:**
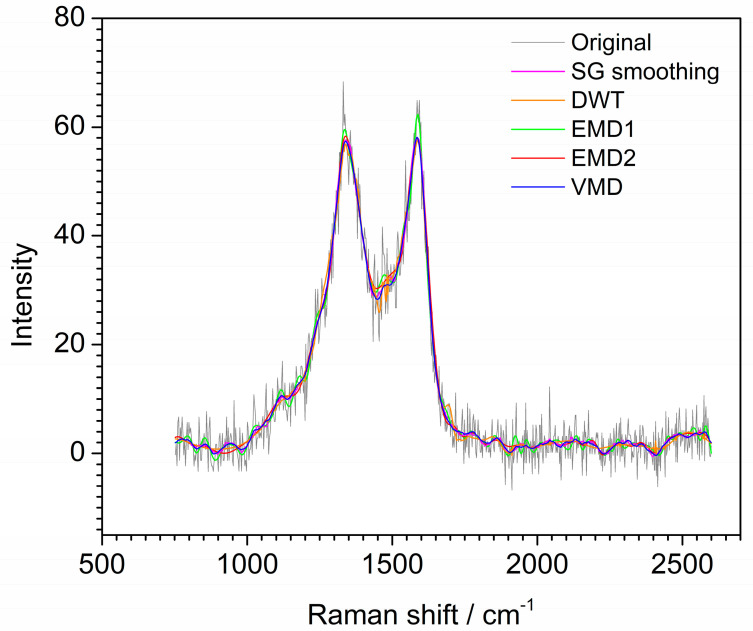
Denoised signals by different methods for the Raman spectrum of MnCo-ISAs/CN.

For further comparison, the Raman spectrum of material MnCo-ISAs/CN is also denoised by SG smoothing and DWT. Figure 8 shows the denoised signals by SG smoothing, DWT, EMD and VMD. For SG smoothing, the window size is selected as 33. The spectrum located at 1500 cm^−1^ still contains noise. For the denoising result of DWT, the denoised spectrum is smoother than that of SG smoothing. However, the peak located at 1350 cm^−1^ is not smooth enough. Moreover, the spectrum located at 1500 cm^−1^ still contains noise after DWT denoising. For the result of EMD, IMF5–IMF8 and **r** are reconstructed to obtain the Raman denoised spectrum and IMF1–IMF3 are removed as noise. However, IMF4 can be served as noise mode and also be served as useful information mode due to it is a mixing mode. If IMF4 is used to reconstruct the denoised spectrum as a useful information mode, the result is labeled as EMD1 and shown in Figure 8. The denoised spectrum still contains much noise and the endpoint effect has appeared at the right end. Moreover, for the result of EMD1, the peaks at 1350 cm^−1^ and 1600 cm^−1^ of the denoised spectrum are higher than that of other methods. If IMF4 is removed as noise mode, IMF5–IMF8 and **r** are used to reconstruct the denoised spectrum for EMD and labeled as EMD2 in Figure 8. It is obvious that the denoised spectrum is smoother than that of when IMF4 is reserved as useful information mode. However, the weak peak in the range of 1400–1700 cm^−1^ is removed. As shown in Figure 8, the spectrum denoised by EMD has an endpoint effect, resulting in spectrum distortion at right end. Furthermore, some useful information after denoising is lose due to mode mixing. For the result of VMD, u1 and u2 are reconstructed to obtain the Raman denoised spectrum, u3–u6 are removed as noise. VMD shows excellent denoising capability and almost all useful information is retained. Moreover, the spectrum denoised by VMD is smoother than that of EMD. Hence, it can be concluded that the denoising effect of VMD is better than that of SG smoothing, DWT, and EMD for the Raman spectrum of MnCo-ISAs/CN.

### 2.4. Denoising of the Raman Spectrum of Fe-NCNT

**Figure 9:**
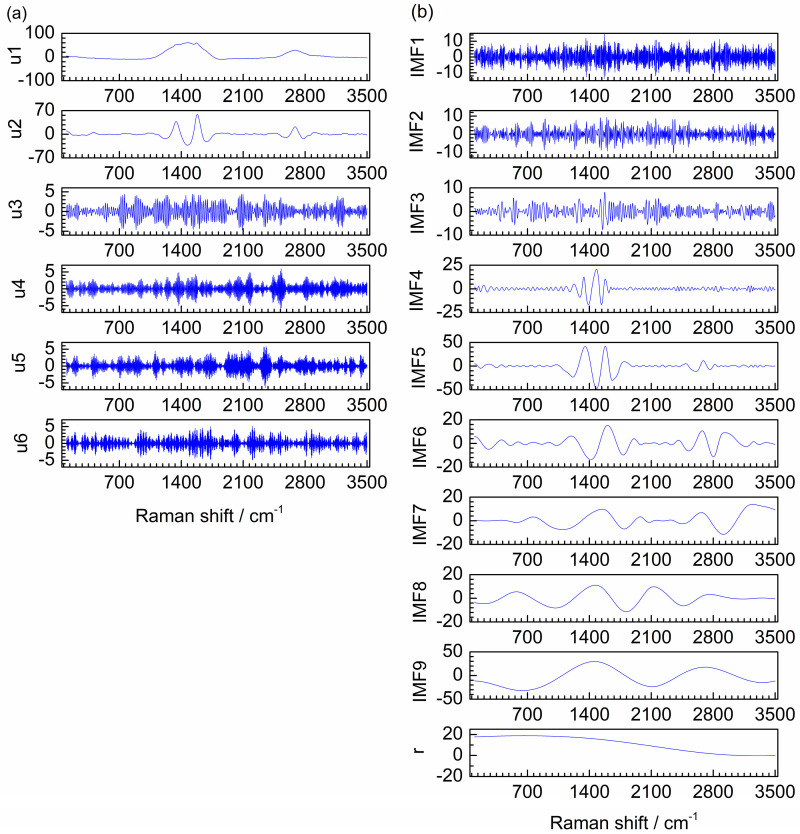
Decomposition results of VMD (**a**) and EMD (**b**) for the Raman spectrum of Fe-NCNT.

To further verify the proposed method, another Raman spectrum of iron-loaded carbon nanotubes (Fe-NCNT) was denoised by VMD. For the decomposition result of VMD, u1–u6 are obtained, which are shown in Figure 9a. It is clear that u1 and u2 are the low-frequency modes and u3–u6 are the high-frequency modes. The different frequency modes are obtained without mode mixing by VMD. Hence, this actual Raman spectrum also indicates VMD is easy to distinguish the boundary between noise modes and useful information modes.

EMD is also used to compare the denoising effect of the proposed method for Raman spectrum of Fe-NCNT. For the decomposition result of EMD, IMF1–IMF9 and a **r** are obtained, which are shown in Figure 9b. IMF1–IMF3 are high-frequency modes obviously and IMF5–IMF9 and **r** are low-frequency modes. IMF4 is low frequency in the range of 1100–1700 cm^−1^ and high frequency in other ranges. Thus, IMF4 is a mixing mode. Therefore, the modes obtained by EMD have mode mixing obviously.

**Figure 10:**
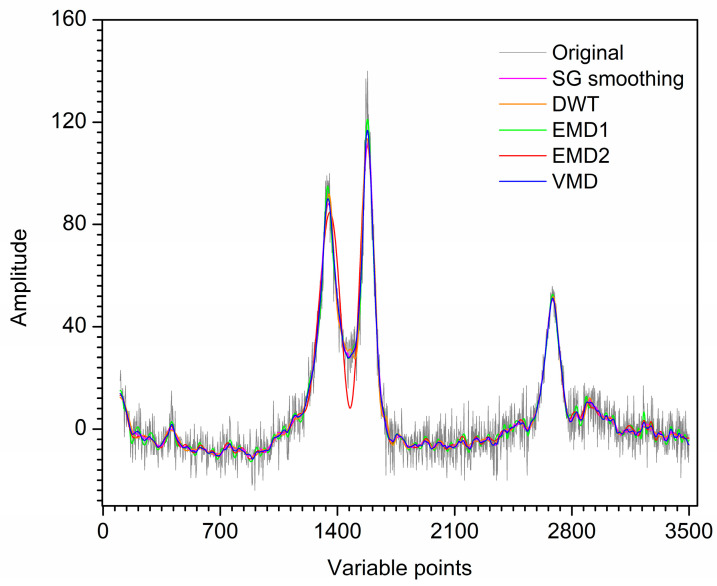
Denoised signals by different methods for the Raman spectrum of Fe-NCNT.

For further comparison, SG smoothing and DWT are also applied to the Raman spectrum of Fe-NCNT. The denoised spectra by SG smoothing, DWT, EMD and VMD are shown in Figure 10. For SG smoothing, the window size is selected as 41. For EMD, since IMF4 is a mixing mode, it can be regarded as noise mode and also be regarded as useful information mode. If IMF4 is used to reconstruct the denoised spectrum, the result is labeled as EMD1 as shown in Figure 10. If IMF4 is removed as noise, the result is labeled as EMD2 in Figure 10. For the decomposition result of VMD, u1 and u2 are used to reconstruct the denoised spectrum and u3–u6 are removed as noise. It is obvious that most noise has been removed by SG smoothing, DWT, EMD1, EMD2 and VMD. The spectrum denoised by VMD is smoother than that of SG smoothing and EMD1. Moreover, the denoising result of VMD improves mode mixing generated by EMD. For the result of EMD2, the denoised spectrum is distorted in the range of 1100–1750 cm^−1^ since the existence of mode mixing. For the denoised spectrum of DWT, the valley around 1500 cm^−1^ still contains noise. The peaks at 1300 cm^−1^ and 1600 cm^−1^ of the spectrum by SG smoothing, DWT and VMD are basically coincident. However, for the denoising result of EMD1, the above two peaks are significantly higher than that of other methods. For the small peak located at 400 cm^−1^, it is difficult to detect a small peak due to a large amount of noise in the original spectrum. However, the Fe-based peak of the signal located at 400 cm^−1^ is clearly observed after denoising. The spectrum retains the shape of the peak after VMD, EMD1 and EMD2. However, the small peak deviates from the original position after SG smoothing and DWT. Although the peak after VMD denoising is slightly wider than that of the original signal, the peak after DWT is wider than that of other denoising methods. Therefore, the denoising effect of the proposed method is more ideal than that of SG smoothing, DWT and EMD for the Raman spectrum of Fe-NCNT.

## 3. Methods

### 3.1. Denoising by Variational Mode Decomposition (VMD)

**Figure 11:**
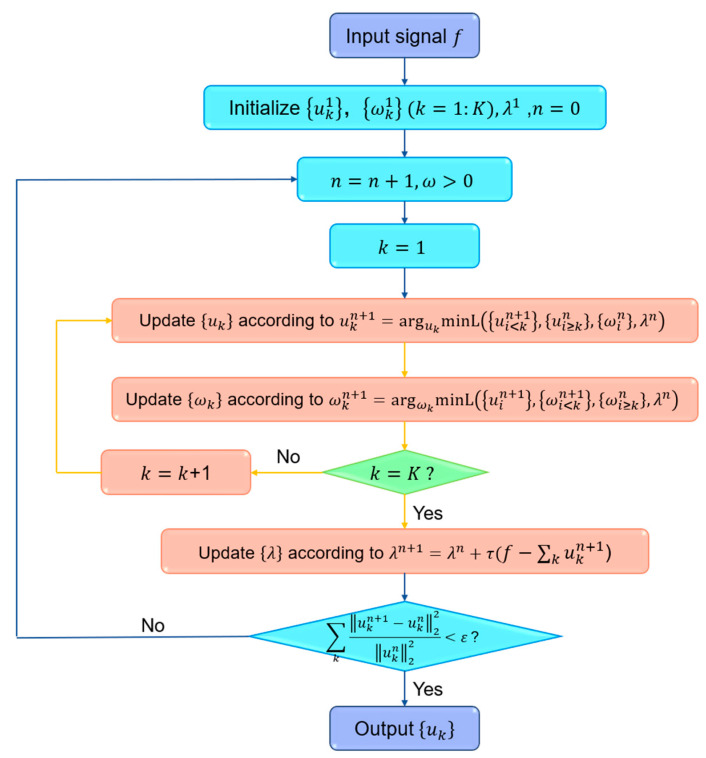
Schematic of the VMD method.

VMD is a decomposition method for adaptive non-recursive signals and a process of solving variational problems. The advantage of this method is that it has solid mathematical theory foundation and strong noise suppression ability. The original signal is decomposed into a series of modes uk, each mode is tight around a central frequency and with limited bandwidth. The sum of all modes is equal to the original signal. The bandwidth problem of modes is transformed into a constrained optimization problem for solving each mode. The above problem is solved by introducing a quadratic penalty term α and Lagrangian multiplier λ. The saddle point of the augmented Lagrangian function is calculated by alternate direction method of multipliers (ADMM). Finally, the final mode uk is obtained by Fourier transform of the analysis signal. The detailed iteration process is shown in Figure 11. The detailed algorithm please refers to reference [31].

In this research, VMD is first introduced for denoising of Raman spectra. The spectrum is decomposed into a series of uk by VMD, all the frequencies of the modes are sorted from low to high. The high-frequency modes represent noise and low-frequency modes represent useful information. After the high-frequency modes are removed, the low-frequency modes are reconstructed to obtain the denoised spectrum.

### 3.2. Denoising by Empirical Mode Decomposition (EMD)

EMD was proposed by Huang et al. in 1998 [22], which is an adaptive decomposition method for non-linear and non-stationary signals. The decomposition of EMD is actually a sifting process [23]. First, all local maxima of the original signal are fitted by cubic spline function to form the upper envelope of the original signal. Similarly, the lower envelope is fitted through all the local minima of the original signal. Then, the mean values **m** of the two envelops are calculated. The first component **h** is obtained by subtracting **m** from the original signal. Whether **h** is an IMF component depends on if it satisfies two conditions defined by IMF. One is that at any point, the mean value is zero of the upper and lower envelopes. The other is that the number of extreme and zero-crossings must be the same or differ by one in the whole data. If **h** does not meet, then **h** is used as the new input signal to perform the above operations until an IMF is obtained. After IMF is obtained, the residual **r** is obtained by subtracting this component from the original signal and determine whether the **r** is a monotone function. Finally, the sifting process is end when the residue does not exceed an extreme value [24]. Based on the inherent characteristics of the signal, the original signal is decomposed into a series of IMFs and a **r** by EMD. The oscillation frequencies of the modes decrease as the order of IMFs increases. After the decomposition, the high-frequency modes are removed as noise and the remaining modes are reconstructed to obtain the denoised signal. As comparison, EMD is also used for the two artificial signals and two actual Raman spectra.

### 3.3. Denoising by SG Smoothing

SG smoothing is a widely used denoising method in spectra analysis. Thus, the method is also used to compare with VMD. SG smoothing is polynomial least square fitting of the data in the moving window. It requires selecting a window with a certain width and an odd number of wavelength points. The center point of the window is replaced with the polynomial least squares fitting values of all points within the selected window [42]. In actual, SG smoothing is essentially a weighted average method with more emphasis on the role of the central point. A symmetric window with length of *i* = 2*w* + 1 is used, where *i* is window size and *w* is the half window width. SG smoothing reduces the influence of randomly changing noise signals by following the shape of the spectrum.

### 3.4. Denoising by DWT

Wavelet transformation is one of the most widely used signal process method in analytical chemistry [43], which includes continuous wavelet transform (CWT) and discrete wavelet transform (DWT). DWT denoising preserves the shape of the original signal. Therefore, DWT is also applied as a comparative denoising method to this research. DWT implements the wavelet transformation by discrete translation factor and telescopic factor. In DWT, the decomposition is achieved through the translation and scaling of wavelet functions. There are tens of wavelet basis functions and numerous decomposition scales can be selected. By DWT, a series of approximations and details is obtained. The noise in signal is removed by processing the details with the hard or soft thresholding strategy. Finally, the processed wavelet coefficients are reconstructed to obtain the denoised signal.

The corresponding matlab codes are used in-house, and the codes are available when there are requirements.

### 3.5. Evaluation of Denoising Performance

In order to evaluate the denoising performance of the method, the figure of spectra before and after denoising and SNR are used. SNR is defined as

(1) $$SNR=10lg\left\{\sum_{s}f^{2}(x)/\sum_{s}{\left[\tilde{f(}x)-f(x)\right]}^{2}\right\}$$

where f(x) is the original signal, $\tilde{f(}x)$ is the denoised signal and *s* is the number of variable points [44]. A larger SNR means less noise mixed into the signal, and a smaller SNR means more noise mixed into the signal. Therefore, the SNR should be as high as possible.

## 4. Datasets

In order to verify the effectiveness of the proposed method, two artificial noised signals and two actual Raman spectra of inorganic materials were employed.

### 4.1. The First Artificial Noised Signal

The first artificial signal was constructed by three different frequency functions to verify the effect of VMD in decomposes the signal into different frequencies. It contains three cosine functions of different frequencies with a random noise, the three cosine functions are expressed by Equations (2)–(4). Figure 1a–e show the first artificial noised signal, functions of f_1_, f_2_, f_3_ and random noise, respectively.

(2) $$f_{1}=\cos\left(2\pi \times 2x-3\right)$$

(3) $$f_{2}=\frac{1}{4}\cos\left(2\pi \times 20x\right)$$

(4) $$f_{3}=\frac{1}{6}\cos\left(2\pi \times 50x\right)$$

### 4.2. The Second Artificial Noised Signal

Due to the characteristic peaks in Raman spectra are the Lorentz peaks, the second artificial signal is constructed by the Lorentz function. The pure signal of artificial signal is constructed by three Lorentz functions which have different peak widths and peak heights and a background function. The three Lorentz functions and the background function are expressed by Equations (5)–(8). The white Gaussian noise with 20 dB is added to the pure signal to obtain the second artificial noised signal. Figure 4a,b show the artificial noised signal and the curves of f_4_, f_5_, f_6_, f_7_, respectively. The artificial noised signal can be considered as artificial Raman noised spectrum, which is composed of 551 variables with a digitization interval of 0.1. As shown in Figure 4a, it is obvious that the artificial signal contains a lot of noise.

(5) $$f_{4}=\frac{2}{\left({\left(5x-70\right)}^{2}+3\right)}$$

(6) $$f_{5}=\frac{5}{\left({\left(x-3\right)}^{2}+3\right)}$$

(7) $$f_{6}=\frac{4}{\left({\left(x+2\right)}^{2}+4\right)}$$

(8) $$f_{7}=0.03\times x^{1/2}$$

### 4.3. The First Actual Raman Spectrum

**Figure 12:**
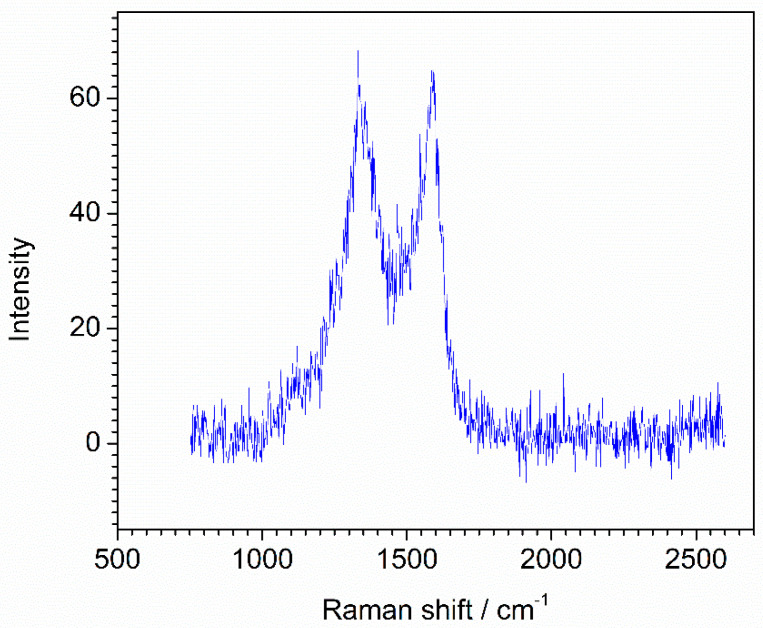
Original Raman spectrum of MnCo-ISAs/CN catalyst.

The first actual Raman spectrum of MnCo-ISAs/CN was measured on a Raman spectrometer (XploRA PLUS, Horiba Kyoto, Japan). The spectrum was recorded with a digitization interval of 2.9 cm^−1^ in the range of 750–2600 cm^−1^, in total of 715 variables. Figure 12 shows the original Raman spectrum of MnCo-ISAs/CN, from which a large amount of noise can be seen. Hence, it is necessary to remove noise and retain valid information.

### 4.4. The Second Actual Raman Spectrum

**Figure 13:**
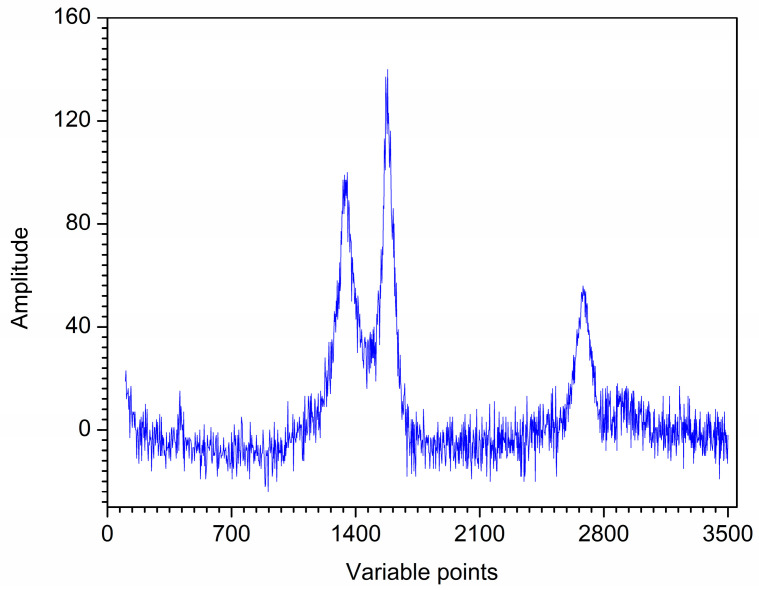
Original Raman spectrum of Fe-NCNT.

The second actual Raman spectrum of the iron-loaded carbon nanotubes (Fe-NCNT) synthesized by a convenient thermal treatment method was measured on a Raman spectrometer (XploRA PLUS, Horiba Kyoto, Japan). The spectrum was recorded with a digitization interval of 3.2 cm^−1^ in the range of 100–3500 cm^−1^, in total of 1351 variables. The Raman spectrum was shown in Figure 13.

## 5. Conclusions

The VMD method was firstly introduced for denoising of Raman spectra with noise. Two artificial noised signals and two actual Raman spectra of inorganic materials were used to verify the denoising performance of the proposed method. The spectrum is decomposed by VMD into a series of modes, which are sorted from low frequency to high frequency. The high-frequency modes are removed and the low-frequency modes are reconstructed to obtain denoised spectrum. In order to verify the denoising effect of this method, SG smoothing, DWT and EMD are used as comparisons. Results show that VMD has highest SNR among the four denoising methods for the two artificial signals. Compared with the denoised result of EMD, VMD can decompose the signal into different modes with different frequencies, which improves the mode mixing and endpoint effects. Moreover, it is easier to distinguish the noise modes and useful information modes by VMD than that of EMD. The peaks of the spectrum denoised by VMD are smoother than those by SG smoothing and DWT. For the small sharp peaks, some information is lost after VMD denoising. However, SG smoothing, DWT and EMD lose more information than that of VMD. Hence, the proposed method shows effectiveness both in SNR and denoising performance compared with SG smoothing, DWT and EMD. Therefore, VMD offered a promising tool for signal denoising in Raman spectral analysis of inorganic materials. In this research, the Raman spectra of two inorganic materials, i.e., MnCo ISAs/CN and Fe-NCNT, were mainly used. We will further explore the Raman spectral denoising approach for organic polymers in the future.

## Data Availability

Not applicable.
